# Use of Biocarbon and its Agglomerates in Silicon and Ferrosilicon Production

**DOI:** 10.1021/acsomega.6c02207

**Published:** 2026-09-23

**Authors:** Tin Tin Kyaw, Merete Tangstad

**Affiliations:** Department of Materials Science and Engineering, Norwegian University of Science and Technology, 7034 Trondheim, Norway

## Abstract

This paper reviews the use of biocarbon as a renewable carbon source to reduce CO_2_ emissions in silicon and ferrosilicon production. Fundamental methodologies for characterizing biocarbon properties and their key findings are discussed and summarized. Charcoal, as a renewable reductant, has demonstrated promising potential to replace fossil carbon because of its thin cell walls and high porosity, which enhance its reactivity toward SiO gas. In addition, both charcoal and SiC-converted charcoals possess lower electrical conductivity compared with conventional carbon materials. However, a significant amount of fines is also generated during the processing and handling of biocarbon materials. The complete (100%) replacement of biocarbon still presents challenges for operations in the submerged arc furnace, as these fines reduce gas permeability and lead to uneven pressure distribution in the furnace. Rather than being discarded, these fines can be reused through densification methods that improve their mechanical strengths. In the production of charcoal agglomerates, selection of an appropriate binder is crucial. Therefore, the performance of charcoal briquettes produced with different types of binders is also reviewed. Improving the mechanical strength of charcoal briquettes/pellets, while achieving optimal mechanical strength and gas reactivity, is crucial for their effective use as biocarbon reductants in silicon and ferrosilicon production.

## Graphical abstract

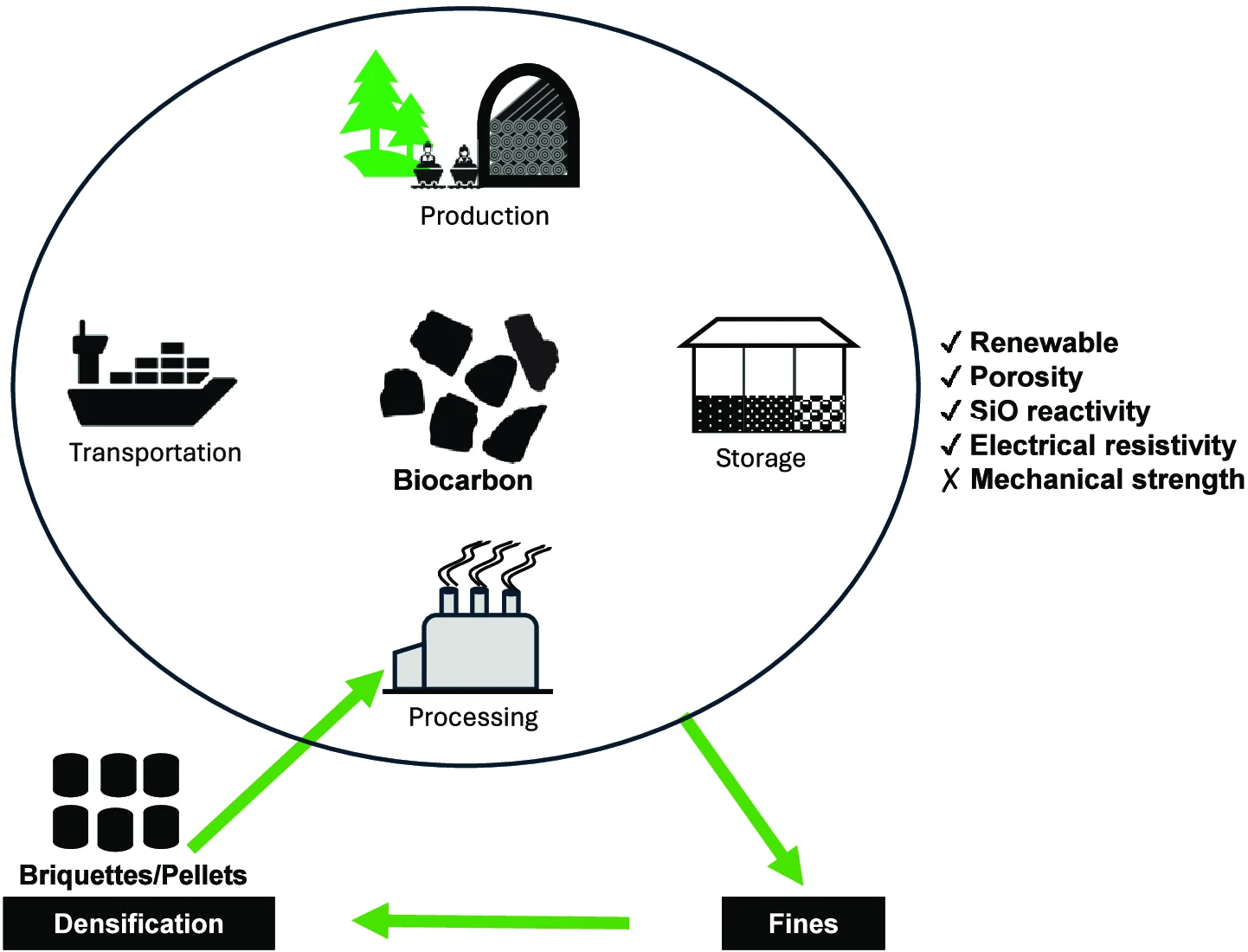

## 1 Introduction

The worldwide production of both silicon and ferrosilicon reached approximately 9,700,000 metric tons in 2024.^1^ The demand for silicon in solar photovoltaics is projected to increase to 675,000– 808,000 tons by 2040.^2^ Silicon and ferrosilicon are widely utilized as alloying elements in aluminum alloy production and steelmaking.^3^ The use of silicon, in the form of ferrosilicon (FeSi) and ferrosilicon–manganese (FeSiMn), enhances the mechanical properties of steels and aluminum alloys. 45% of silicon is consumed in aluminum alloy production, followed by 35% used in the production of silicones and silanes.^4^ In cast iron production, ferrosilicon performs as an inoculant to promote graphite nucleation.^3^ High-purity silicon is utilized in the chemical industry (silicones and silicanes), medical industry (electric insulators, protective coatings) and electronic industry (semiconductors and solar photovoltaic devices).^3^

Silicon and ferrosilicon are produced by the carbothermic reduction of quartz with carbonaceous reductants in a submerged arc furnace (SAF).^5^ The difference is that an iron source is added to the charge in ferrosilicon production.^3^ The chemical reactions in the furnace are believed to be similar in both silicon and ferrosilicon production. The reactions are typically divided into two temperature zones.^5^ In the low temperature zone (700–1800 °C), iron is reduced according to reaction [Disp-formula eqR.1]. Silicon carbide (*SiC*_*s*_) forms when carbon reacts with silicon monoxide gas (*SiO*_*g*_), as shown in reaction [Disp-formula eqr2]. In the high temperature zone in the lower part of the furnace (1800– >2000 °C), reactions [Disp-formula eqR.3] and [Disp-formula eqR.4] involve SiO gas generation from the reactions of silica with silicon carbide, as well as silica with silicon, respectively. Finally, silicon is produced through the reaction of silicon carbide (*SiC*_*s*_) with silicon monoxide gas (*SiO*_*g*_) (reaction [Disp-formula eqR.5]).

**Low-temperature zone (700–1800 °C)**

R.1 $${\mathrm{Fe}}_{2}O_{3}\left(s\right)+3\mathrm{CO}\left(g\right)=2\mathrm{Fe}+3CO_{2}\left(g\right),{\Delta H}_{1200{}^{\circ}C}=-10.28\;\mathrm{kJ}$$

R.2 $$\begin{array}{cc} \mathrm{SiO}\left(g\right)+2C\left(s\right)=\mathrm{SiC}\left(s\right)+\mathrm{CO}\left(g\right) & {\Delta H}_{1500{}^{\circ}C}=-21.71\;\mathrm{kJ} \end{array}$$

**High-temperature zone (1800– >2000 °C)**

R.3 $$\begin{array}{cc} 2\mathrm{Si}O_{2}\left(s,l\right)+\mathrm{SiC}\left(s\right)=3\mathrm{SiO}\left(g\right)+\mathrm{CO}\left(g\right) & {\Delta H}_{1900{}^{\circ}C}=1382.9\;\mathrm{kJ} \end{array}$$

R.4 $$\begin{array}{cc} \mathrm{Si}O_{2}\left(s,l\right)+\mathrm{Si}\left(l\right)=2\mathrm{SiO}\left(g\right) & {\Delta H}_{1900{}^{\circ}C}=654.66\;\mathrm{kJ} \end{array}$$

R.5 $$\begin{array}{cc} \mathrm{SiC}\left(s\right)+\mathrm{SiO}\left(g\right)=2\mathrm{Si}\left(l\right)+\mathrm{CO}\left(g\right) & {\Delta H}_{1900{}^{\circ}C}=171.32\;\mathrm{kJ} \end{array}$$

Greenhouse gas emissions from metallurgical grade silicon (MG-Si) production arise from the carbothermic reduction of quartz, energy consumption, mining, and transportation of raw materials. Total global emissions from the MG-Si production are approximately 12 metric tons of CO_2_-equivalents (CO_2_e) per ton of MG-Si.^6^ In Norway, the industry and mining sector accounted for 10.6% of the country’s total emissions in 2024.^7^ Aligned with the Paris Agreement adopted in 2015, which aims to limit global warming to below 2 °C, Norway has committed to reducing its greenhouse gas emissions by at least 40% relative to 1990 levels (52 Mt CO_2_e) by 2030.^8^ Electricity in Norway comes from renewable sources such as hydropower. Thus, CO_2_ emissions are primarily associated with the carbothermic reduction processes, with 4.7–5 t CO_2_e/t Si and 3.5–4.6 t CO_2_e/t FeSi.^9,10^ Possible pathways for reducing CO_2_ emissions are the use of renewable biocarbon, carbon capture and utilization and storage (CCU/CCS), metallothermic reduction, use of hydrogen, and electrolysis.^11^ Although biocarbon, CCU and CCS have already been applied at the industrial scale, hydrogen-based and electrolysis reductions remain at early stages of research.^11,12^

The use of biocarbon does not fully eliminate the carbon emissions. However, it can approach carbon neutrality by offsetting carbon emissions absorbed by plants during their lifetime.^11,12^ Studies have shown that charcoal, as a renewable biocarbon, possesses metallurgical properties comparable to those of fossil carbon. Nevertheless, it still has limitations, particularly, its poor mechanical properties. Thus, this paper aims to review not only fundamental characterizations of biocarbon properties but also densification methods used to improve the mechanical strengths of biocarbon agglomerates. In addition, charcoal briquettes prepared with different types of binders, along with the corresponding findings, are also discussed and summarized.

## 2 Carbon Reductants

The carbon materials used in silicon and ferrosilicon production are classified into fossil carbon (coal, coke and char) and renewable carbon (charcoal and woodchips).^13–22^

Coal is a readily combustible rock that consists of more than 50% by weight and 70% by volume of carbon-rich materials.^23^ It is formed through the compaction and hardening of plant remains such as peaty deposits that have undergone varying degrees of alteration. Coal is composed of different macerals, which include vitrinite, liptinite, inertinite, and minerals. During carbonization, vitrinite and liptinite function as reactive macerals that form the binder phase, whereas inert macerals remain unchanged and perform as the filler phase.

Coal is characterized by three fundamental properties: coal grade, coal type and coal rank.^24^ Coal grade refers to the level of purity of coal and the amount of mineral matter present in the coal matrix. Coal type depends on the nature of original plant matter from which the coal was formed. Coal rank identifies the extent of coalification or maturation that the coal has undergone.^23–25^ As pressure, temperature and time increase, the rank of coal also rises; for instance, bituminous and anthracite are coals with higher ranks.

Metallurgical coke production involves heating coal within the bituminous range (coking coal) or coal blends in the absence of air under controlled conditions.^26^ Upon heating at 900–1095 °C, certain macerals, such as vitrinite in bituminous coal, soften, become plastic, and fuse into a fluid phase that later solidifies to form a strong coke. Coke properties are characterized by coke grade, type and rank, which are strongly influenced by the original properties of coal.^25^ It is noted that the FeSi/Si producers often use the term coke to refer to semi-coke.

Semi-coke is produced from low-rank coal, such as sub-bituminous coal, through dry distillation and pyrolysis at lower coking temperature than that used for the metallurgical coke, typically around 500-600 °C.^27,28^ During carbonization, a number of volatile matter, sulfur- and nitrogen-containing compounds is released, while tar is separated out. The remaining plastic matter undergoes repolymerization and transforms into semi-coke with a high fixed carbon content.^29^

Char is also produced through the carbonization of coal or biomass, but it does not pass through a fluid stage during carbonization, unlike semicoke or coke.^30^ Char can form when nonbituminous coals are carbonized at extremely high heating rates. The main difference between char and semicoke is the source of carbon, with char derived from either coal or biomass, but semicoke is produced from coal.

Petroleum coke, commonly referred to as pet.coke, is a by-product of the oil refining process known as delayed coking.^31^ During the process heavy fractions from crude oil distillation are heated to around 500 °C, causing thermal cracking that produces lower molecular weight hydrocarbons, and leaves behind a solid residue known as petroleum coke. Considering the coking process and properties, petroleum coke is categorized into different types such as sponge, shot, needle, purge, and catalyst coke.

Charcoal is a renewable carbon that is produced by pyrolysis and carbonization of wood under moderate heating conditions.^32–34^ Upon heating at <110 °C, moisture is first removed from the wood. The wood then decomposes endothermically, releasing water vapor at 110–270 °C. As the temperature increases to 270–400 °C, the structure of wood transforms exothermically with the release of volatile gases and acetic acid and tar. At 500 °C, more tar is decomposed, resulting in the increase of the fixed carbon content and the decrease in the volatile matter.^32^ The charcoal undergoes further gasification and degradation at 500–1000 °C.

Woodchips are porous and irregular in shape, creating voids in the burden in the furnace, which increase gas permeability, thereby reducing the effects of blowing.^35^ The internal report of carbonaceous reductants used in the silicon process in 2001 in Figure 1 indicates that woodchips are commonly added to the reduction process.^25,36^

**Figure 1:**
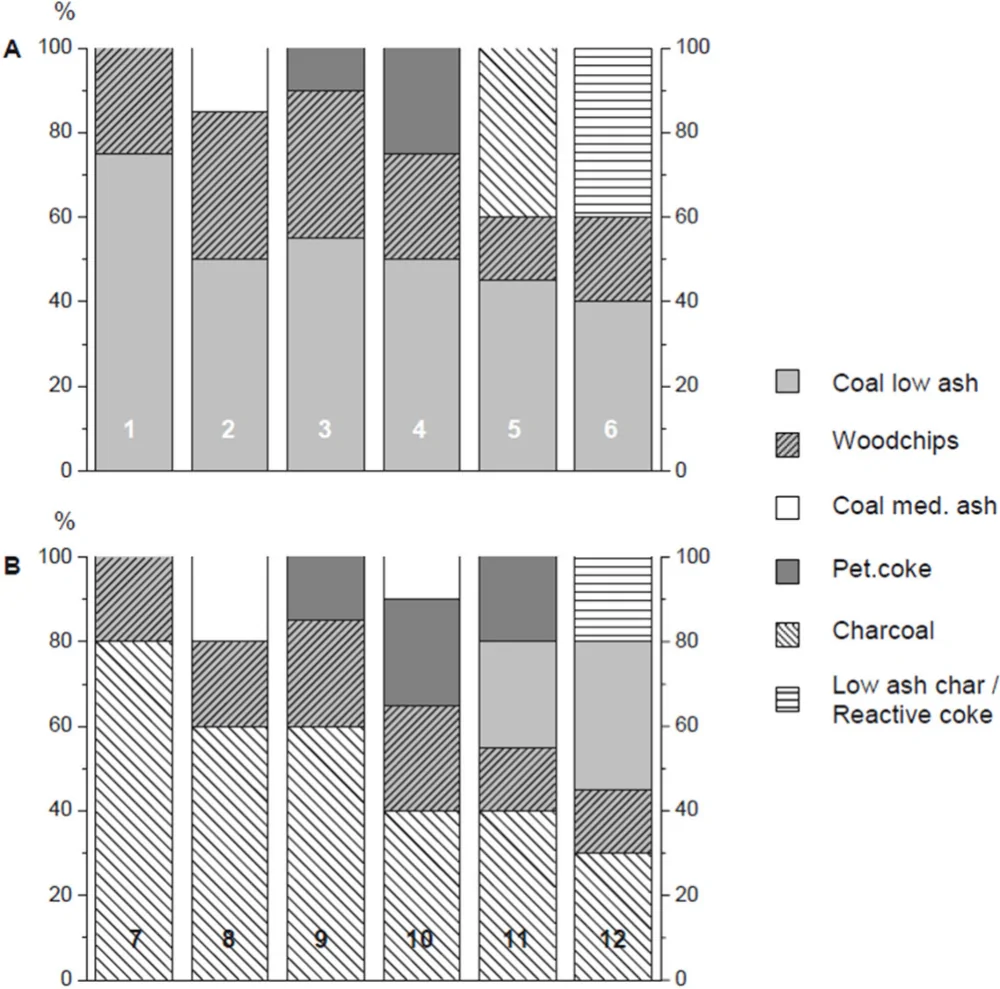
Usage of carbonaceous materials as reductants in the silicon process across different regions worldwide based on (A) fossil carbon and (B) charcoal.^36^ Reproduced with permission from ref 25. Copyright 2008 Norwegian University of Science and Technology.

### 2.1 Properties of Carbon Reductants

The selection of carbon reductants in silicon production must take into account several critical factors.^13–21^ These include proximate analysis, structural characteristics, SiO reactivity, mechanical strength, thermal strength, and electrical resistivity. Chemical specifications of these carbon reductants are expected to have low ash content, minimal volatile matter, and high fixed carbon. Additionally, economic feasibility and materials availability are important considerations.

#### 2.1.1 Proximate Analysis

The composition of carbon materials is typically characterized by proximate and ultimate analyses.^32^ Proximate analysis of carbon materials provides moisture content, volatile matter, ash, and fixed carbon (fix C) content, whereas ultimate analysis determines the elemental composition (C, H, N, O, S, P).^37^

Moisture, which consists of water composed of hydrogen and oxygen, occurs in two forms: surface moisture and inherent moisture.^15,38^ Surface moisture refers to water present on the exterior surface of the carbon material, while inherent moisture is retained within the pores and capillaries of the material. According to ASTM D3173/D3173M-17a,^39^ 1 g of sample is heated in an oven with dry air circulation at 104–110 °C for 1 h. The moisture is calculated by the following eq 1, where m_i_ is the initial mass of sample before heating, and m_f_ is the final mass of sample after heating.

1 $$\mathrm{moisture}\;\left(\%\right)=\frac{\left(m_{i}-m_{f}\right)}{m_{i}}\times 100$$

Volatile matter is composed of aliphatic hydrocarbon atoms (acyclic and cyclic) and aromatic carbon compounds (mono-ring and multi-ring), phenolics, and O-compounds.^40^ According to ASTM D3175-20,^41^ volatile matter is measured by heating 1 gram dried sample at 950±20 °C for 7 min in the absence of air. The volatile matter is then calculated using the mass before heating (m_i_) and after heating (m_f_), after the removal of moisture (eq 2).^41^

$$\mathrm{volatiles matter}\;\left(\%\right)=\left(\frac{\left(m_{i}-m_{f}\right)}{m_{i}}\times 100\right)-\mathrm{moisture}\;\left(\%\right)$$ 2

Ash consists of inorganic components such as limestone, silicon, iron, aluminum, clay, quartz, and minor trace elements such as zinc, copper, and boron.^40^ To measure the ash content regarding ASTM D3174–12,^42^ 1 g of the dried sample is placed in a capsule with a cover. The sample is heated to for 750 °C for coal samples and 950 °C for coke samples. The ash content is calculated using the mass of sample before heating (m_i1_), the mass of empty capsule and cover (m_i2_), and the mass of the sample after heating including capsule and ash residue­(m_f_), shown in eq 3.^42^

3 $$\mathrm{ash}\;\left(\%\right)=\frac{\left(m_{f}-m_{i2}\right)}{m_{i1}}\times 100$$

Fix C and ash are the combustible solid residue remaining after volatiles and moisture have been removed.^25^ Carbon present in ash and volatile matter is not considered part of the fixed carbon content. Fix C is calculated by subtracting the contents of volatiles, moisture, and ash according to eq 4. It is noted that Fix C, ash, and volatiles are typically measured on a dry basis, that is after the removal of the moisture.

4 fix C(%)=100−moisture(%)−volatiles(%)−ash(%)

#### 2.1.2 Structural Characteristics

The physical properties of carbon materials are the porosity, density, cell-wall thickness, surface area, and graphitization. These properties are related to the SiO reactivity, mechanical strength, and electrical resistivity of carbon materials.

The porosity of a carbon material is calculated by the absolute density (true density) of solid carbon and apparent density (envelope density), expressed in eq 5.^43^ Absolute density represents the density of the solid carbon excluding open pores, while apparent density accounts for the solid carbon including all pores and surface irregularities.

5 $$\mathrm{porosity}\;\left(\%\right)=\left(1-\frac{\mathrm{apparent density}}{\mathrm{absolute density}}\right)\times 100$$

Kaffash^44^ and Jayakumari^45^ investigated porosity of different carbon materials. The results are summarized in Table 1. Charcoal exhibited lower particle density (0.5–0.56 g/cm^3^) and higher porosity (68–80%) than metallurgical, calcined and petroleum cokes. The density and porosity of charcoal depend on the original wood material and pyrolysis temperature. As the pyrolysis temperature increases up to 800°C, the porosity increases due to the release of volatiles. Simultaneously, the charcoal undergoes shrinkage as a result of the reduction in volume associated with the condensation of the carbon crystallites.^46^ Furthermore, Charcoal with high porosity consists of thin cell walls. The surface area and cell wall thickness of the carbon material are closely related to its porosity. Thus, the combination of high porosity and thin cell wall thickness contributes to the charcoal’s greater reactive surface area.^47^

**Table 1:** Porosity of Carbon Materials^44,45^

sample	density (g/cm^3^)	porosity (%)	ref
charcoal A	0.50	68	^44^
charcoal B	0.48	80	^44^
charcoal C	0.56	74	^44^
charcoal		75	^45^
met. coke	0.9–1.1	44–55	^48^
calcined coal		55	^45^
pet. coke		20	^45^

Carbon materials undergo graphitization at high-temperature conditions during silicon production, gradually converting from its initially disordered microcrystalline carbon layer structure into a more ordered structure.^49^ The graphitization index defines how much a carbonaceous material resembles the atomic structure of ideal graphite. Common characterization methods for graphitization are X-ray diffraction (XRD), Raman spectroscopy and Fourier-transform infrared (FTIR).^50^ Tao et al.^50^ studied the graphitization behavior of various carbonaceous materials (bituminous coal, charcoal, non-caking coal, and weak-caking coal) within the temperature range of 1300–1600 °C. Each material reaches its graphitization onset at different temperatures, 1982 °C for bituminous coal, 2457 °C for charcoal, 2097 °C for non-caking coal, and 1889 °C for weak-caking coal. Charcoal, due to the presence of alkali metal and its porous structure, enhances chemical reactivity, which suppresses its degree of graphitization with increasing temperature. In contrast, the graphitization degree in bituminous coal, non-caking coal and weak-caking coal increases with the temperature, attributed to their low alkali metal content, which promotes microcrystalline structural changes within the organic phase.

#### 2.1.3 SiO Reactivity

The ability of carbonaceous materials to react with *SiO*_*g*_ and form SiC­(reaction [Disp-formula eqr2]) is referred to as SiO reactivity.^51^ The conversion of SiC from carbon materials in the low-temperature zone is crucial for improving the silicon production yield. The loss of SiO gas, due to incomplete reactions with carbon materials, reduces the silicon production yield and leads to increased energy consumption.^45^ SINTEF SiO-reactivity test^51,52^ and Elkem SiO-reactivity test^53^ are common methods to evaluate the SiO reactivity of carbon materials.

The SINTEF SiO-reactivity test, shown in Figure 2, was originally developed by Tuset and Raaness in 1974 to evaluate and select suitable carbonaceous reductants in silicon production.^52^ Linstad et al.^51^ later introduced incremental improvements in the test. The reactivity (*R*10) in eq 6 is calculated by the concentration of CO (*Q*_Ar_) in the gas leaving the condensation chamber, which is continuously recorded as a function of time (Δ*t*). A lower R10 value indicates a higher SiO reactivity of carbon materials.

6 $$R10=\underset{18\to 10}{\sum }\frac{Q_{\mathrm{Ar}}\left(18-\mathrm{pctCO}\right)}{82-0.82\times \mathrm{pctCO}}\times \Delta t$$

**Figure 2:**
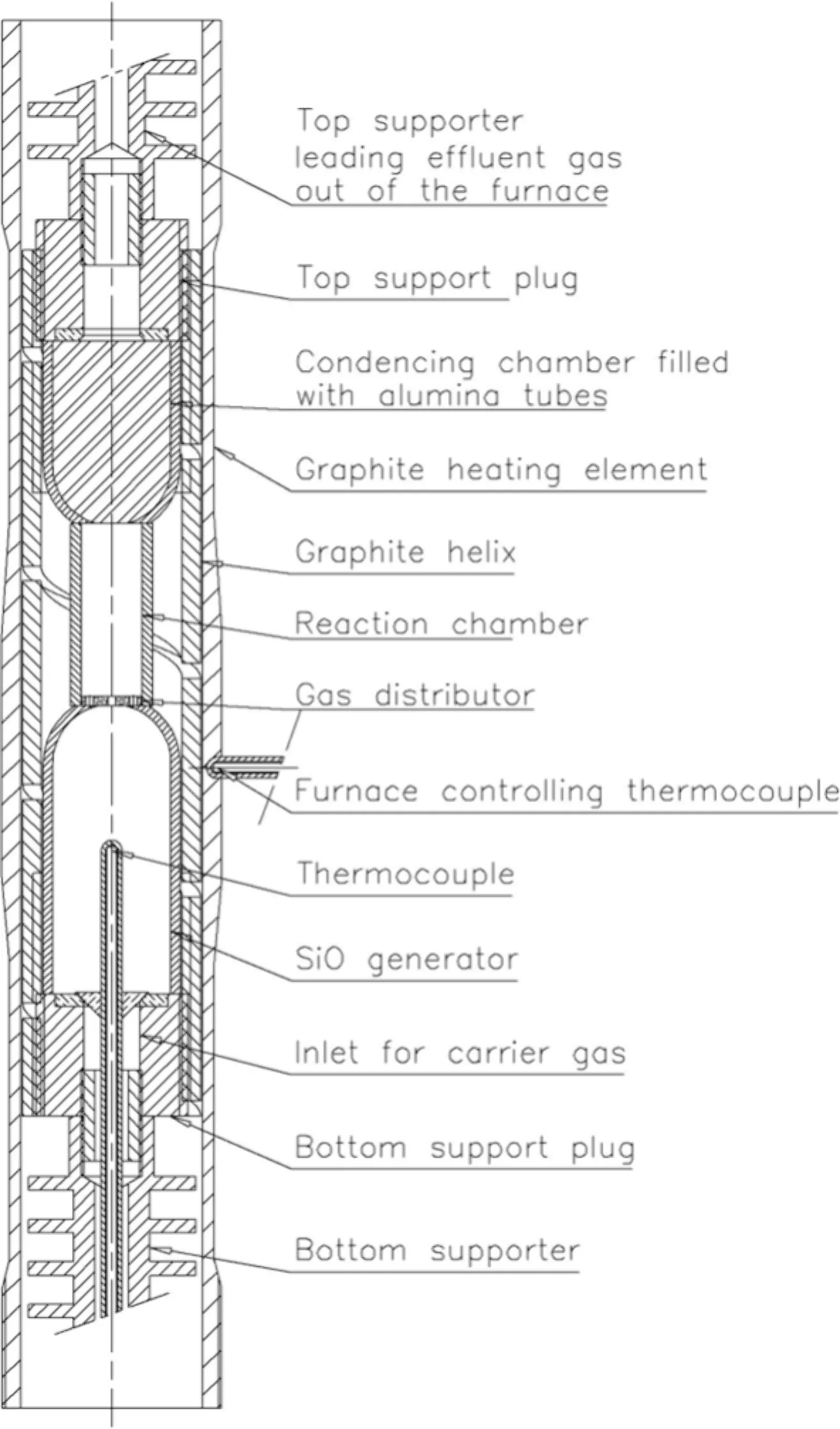
Experimental setup of SINTEF SiO-reactivity test. Reproduced with permission from ref 51. Copyright 2007 Mintek.

Buø^53^ from Elkem developed a method to evaluate the influence of carbon reductants on silicon recovery in silicon and ferrosilicon production using a thermobalance, as illustrated in Figure 3. The SiC conversion of coke particles is calculated by the sample mass loss before and after the experiment. This conversion is an indication of the reaction rate, which is expressed in the ratio of carbon mole reacted to the initial mole of carbon in coke, with respect to the change in time (Δt), as given in eq 7.

7 $$\%\mathrm{C reacted}=\frac{\mathrm{mole C reacted}}{\mathrm{mole C in coke sample}}\times 100$$

**Figure 3:**
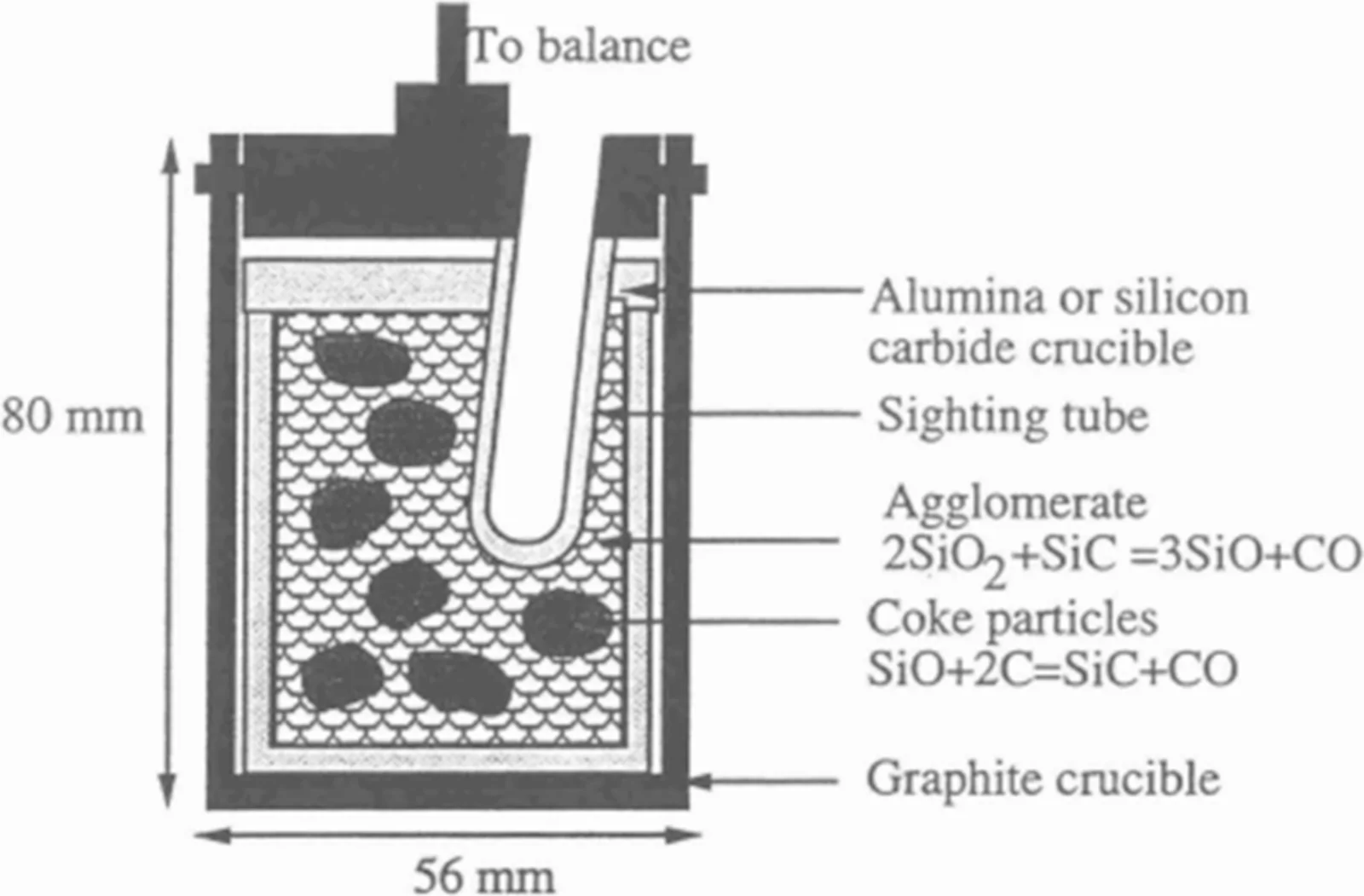
Experimental setup of Elkem SiO-reactivity test. Reproduced with permission from ref 53. Copyright 1995 Mintek.

Several studies have compared the SiO reactivity of traditional fossil carbon and biocarbon such as charcoal. The SiO reactivity of carbonaceous materials is primarily influenced by the properties of the original material source such as the coal rank, distribution of binder and filler phases in the carbonized product.^14^ Raaness et al.,^51^ according to the SINTEF reactivity test, investigated the reactivity of chars prepared from eight different coals, ranging from high volatile bituminous (HVB) coals to medium volatile bituminous (MVB) coals. They reported that the reactivity of chars toward the SiO gas increases as the coal rank or vitrinite reflectance decreases in bituminous coals. This enhanced reactivity is attributed more to the binder phase, which consists of carbon derived from the reactive macerals, rather than the filler phase, which comprises inert organic and mineral matter. In addition, cokes containing isotropic carbon structures were also found to be more reactive than those with anisotropic structures. Similarly, Myrvågnes and Lindstad^14^ observed that the presence of the filler phase reduced reactivity toward SiO gas, whereas the inclusion of inherent clastic quartz grains within the coal matrix increased the SiO reactivity. These findings indicate that coals of similar rank can exhibit different SiO reactivity due to variations in their petrographic and mineralogical characteristics.

Table 2 summarizes the SiO reactivity results of charcoal and coke obtained using the SINTEF reactivity test.^54–56^ Studies by Paull,^54^ Myrhaug^55^ and Monsen et al.^56^ consistently showed lower SiO gas loss for charcoal than coke, indicating higher SiO reactivity of charcoal. In addition, Ramos^57^ compared SiC conversion of charcoal and coke with previous works.^52,58,59^ The results showed that the degree of SiC conversion in charcoal was 40-90%, higher than coke (30-70%).

**Table 2:** SiO Reactivity of Charcoal versus Coke Using SINTEF Reactivity Test^54–56^

SiO reactivity (mL of SiO gas)		.
charcoal	coke	ref
1842–1971	2510–2900	^54^
160–975	900–2110	^55^
200–1100	1500–2000	^56^

According to the Elkem reactivity test, Buø et al.^60^ conducted the reactivity of cokes. The authors found that coke with a higher proportion of binder phases exhibited greater reactivity due to the increase in porosity. The development of porosity was attributed to the plastic behavior of reactive macerals in coal during carbonization, which facilitated the migration of tars and oils through fine pore networks. Simultaneously, gases trapped within the pore structure expand internally, causing the softened coal to bloat and enlarge the pores. Overall, the findings indicate that charcoal possesses superior SiO reactivity and SiC conversion relative to coke.

#### 2.1.4 Cold Strength

The mechanical strength of carbon materials is essential to minimizing fine generation during mining, transportation, furnace charging, and subsequent heating and high-temperature reactions.^61^ Cold strength refers to the ability of the carbon materials to resist breakage and crushing during handling and processing at ambient temperature. Common methods for evaluating the cold strength are tumbling/drum test, compression test, and drop test.^62,63^

The tumbling/drum test evaluates carbon materials’ resistance to abrasion using a rotating drum where particles collide and rub against each other under repeated impacts and friction.^62,63^ Friability (*F*) and tumbling strength index (TSI) are two parameters commonly determined from this test. Friability qualifies fines generation, while tumbling strength reflects the resistance of particles to degradation.^64,65^ High friability corresponds to greater fines generation, which indicates a lower tumbling strength index. Friability is calculated as the ratio of the mass of fines that pass through a mesh of less than 1 or a specified size (*W*_<1mm_) divided by the initial sample mass (*W*_0_), eq 8.^64^ The tumbling strength index (TSI) is calculated as the ratio of mass of the sample with a particle size above 1 mm or a specified size (*W*_+1mm_) to the initial sample mass (*W*_0_), as expressed [Disp-formula eq9].^65^

8 $$F_{<1\mathrm{mm}}\left(\%\right)=\left(\frac{W_{<1\mathrm{mm}}}{W_{0}}\right)\times 100$$

9 $${\mathrm{TSI}}_{+1\mathrm{mm}}\left(\%\right)=\left(\frac{W_{+1\mathrm{mm}}}{W_{0}}\right)\times 100$$

The compression test evaluates the carbon materials’ strength by applying a compressive load on the sample until it fractures.^66^ It indicates the ability of carbon materials to withstand the pressure exerted by the overlying burden in the furnace.^63^ The compressive strength is calculated by the applied load (*F*) and cross-sectional area of the particle (*A*), according to the following eq 10.^67^

10 $$\mathrm{compressive strength}\;\left(N/m^{2}\right)=\frac{F}{A}$$

The drop test measures the tendency of carbon materials to fracture and generate fines when subjected to repeated impacts from a specific height (1.5 or 2 m).^67,68^ The test reflects the ability of the carbon materials to withstand impact loads during handling operations such as transport chutes.^63^ The impact resistance index (IRI) from the drop test is determined from the average number of drops from a 2 m height and the corresponding number of broken pieces produced, given in eq 11.^67^

11 $$\mathrm{IRI}\;\left(\%\right)=\left(\frac{\mathrm{average number of drops}/2m}{\mathrm{average number of pieces}}\right)\times 100$$

A number of studies have investigated the cold strength of charcoal in comparison with coal and coke.^62,66,69^ Ramos and his team^69^ compared the quality of charcoals produced by pyrolysis of Eucalyptus wood with coke using the drum test. In Figure 4, charcoals exhibited higher friability index than coke, indicating a higher amount of fines generation. Jahrsengene et al.^62^ evaluated dusting potential of several industrial charcoal (CC), metallurgical coke (MC) and lab-made biocarbon derived from Norwegian spruce, birch and steam-exploded pellets pyrolyzed at 500°C. The decrepitation of particles smaller than 0.5 mm in both biocarbon and metallurgical coke was 3–7%, lower than the industrial charcoal (11–13%). Kaffash and Tangstad^66^ investigated the compressive strength of raw charcoal and heat-treated charcoals using the compression test at a constant loading rate of 1 mm/min. The compressive strength of the untreated charcoal ranged from 1.5 to 4.6 MPa, whereas heat-treated charcoals showed higher compressive strength (3–7.29 MPa). Compared to the heat-treated metallurgical coke (20–30 MPa), both untreated charcoals and heat-treated charcoals were mechanically weaker. These studies indicate that charcoal is more fragile and generates more fines than coal and coke.

**Figure 4:**
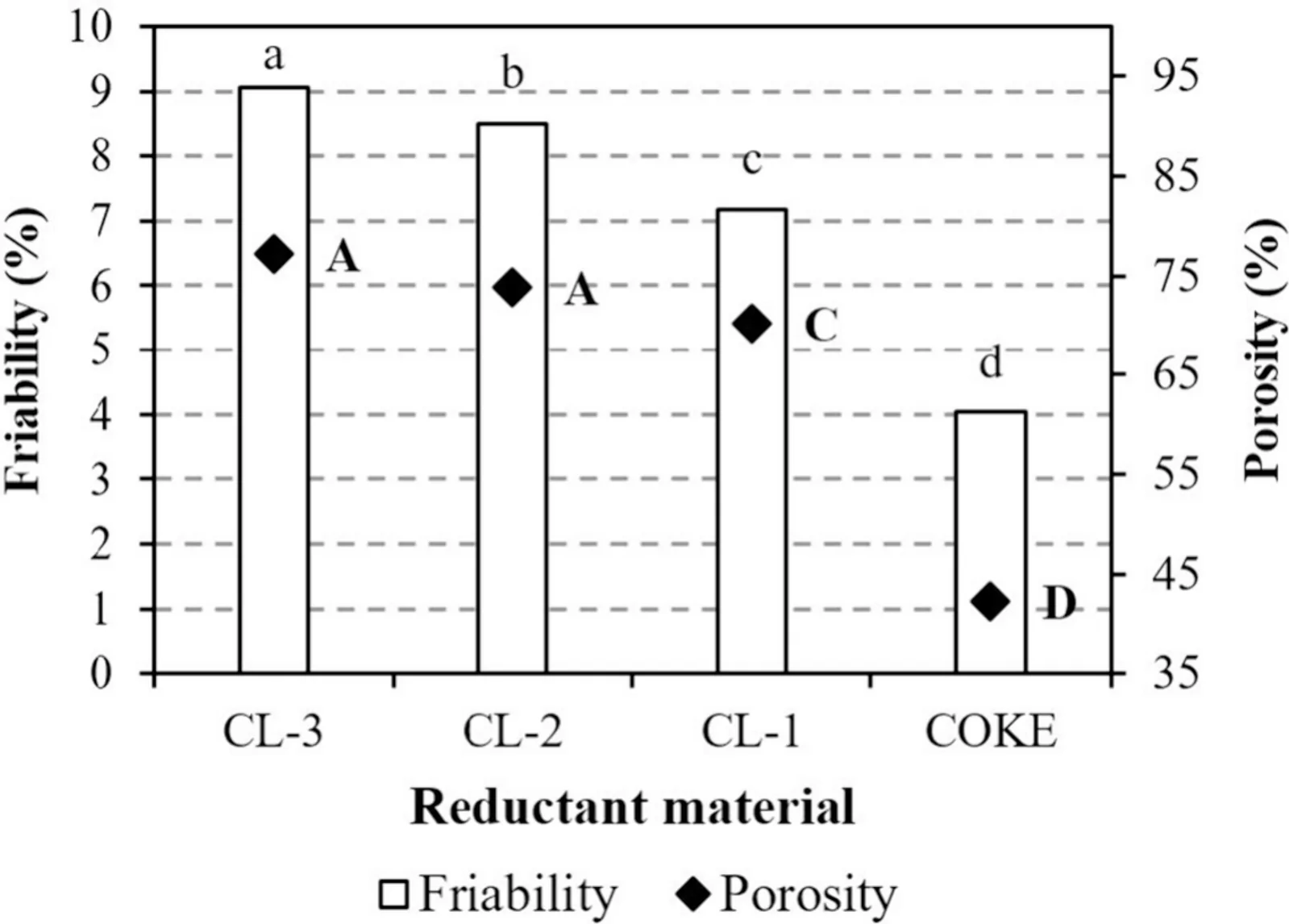
Friability (impact strength) and porosity of charcoals made from Eucalyptus wood (CL) and coke from ref 69. (open access).

#### 2.1.5 Thermal Strength

When carbon reductants react with gases at elevated temperatures in the furnace, their strength is affected by both the temperature and chemical reaction with gases. The thermal strength of carbon materials is commonly evaluated using the CO_2_-reactivity test developed at NTNU/SINTEF, which is based on the reaction between carbon and CO_2_ gas (Boudouard reaction [Disp-formula eqR.6]). Figure 5 illustrates the CO_2_-reactivity test performed in a thermogravimetric furnace under a flowing gas mixture of CO and CO_2_ gases (75%:25% or 50%:50%) at 1000–1100 °C.^70^ The reacted fixed carbon per time is calculated based on the recorded mass measurements during oxidation with CO_2_. As this test examines the gasification behavior of solid carbon through a solid-gas reaction, it indicates the reactivity of carbon materials toward SiO gas. Furthermore, it offers insight into the ability of carbon materials to retain their strength in the upper part of the furnace (800-1000 °C).^19^

R.6 $$\begin{array}{cc} CO_{2}\left(g\right)+C\left(s\right)=2\mathrm{CO}\left(g\right) & {\Delta H}_{1500{}^{\circ}C}=161.77\;\mathrm{kJ} \end{array}$$

After the CO_2_ reactivity test, both the cohesion strength index (C.I.) and thermal strength index (T.I.3) are evaluated by using the tumble/drum test.^70^ While the cohesion strength refers to the ability of carbon materials to maintain their structural integrity during reaction with gases, the thermal strength indicates their resistance to abrasion. The thermal strength is also sometimes termed as the thermal abrasion strength index or thermal stability index.^71,56,70,20^

**Figure 5:**
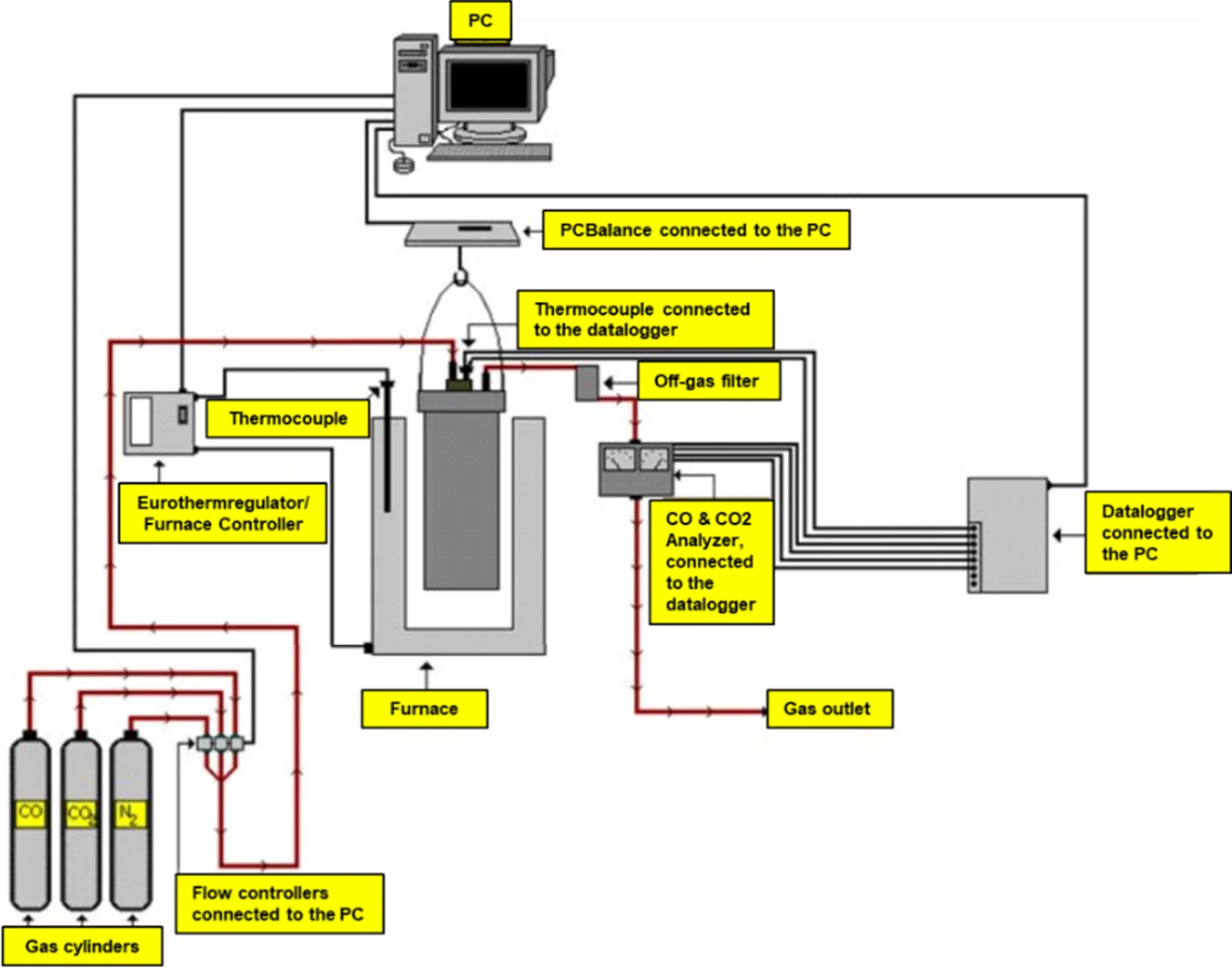
Schematic diagram of CO_2_-reactivity test developed at NTNU/SINTEF. Reproduced with permission from ref 70. Copyright 2007 Mintek.

Monsen et al.^70^ investigated cohesion and thermal abrasion strength of various CO_2_-reacted carbon materials, which is shown in Figure 6. The cohesion strength (C:I) was calculated according to the reacted carbon particles larger than 4.75 mm, whereas the thermal stability index (T.I.3) was evaluated based on the particles larger than 3.3 mm after tumbling (40 rpm and 30 min). Brazilian charcoals exhibited significantly lower cohesion strength (74–84%) and thermal stability index (78–82%) compared to preserved-wood charcoals and metallurgical cokes. Thus, the Brazilian charcoals lost more structural integrity after chemically reacting with gases at high temperatures.

**Figure 6:**
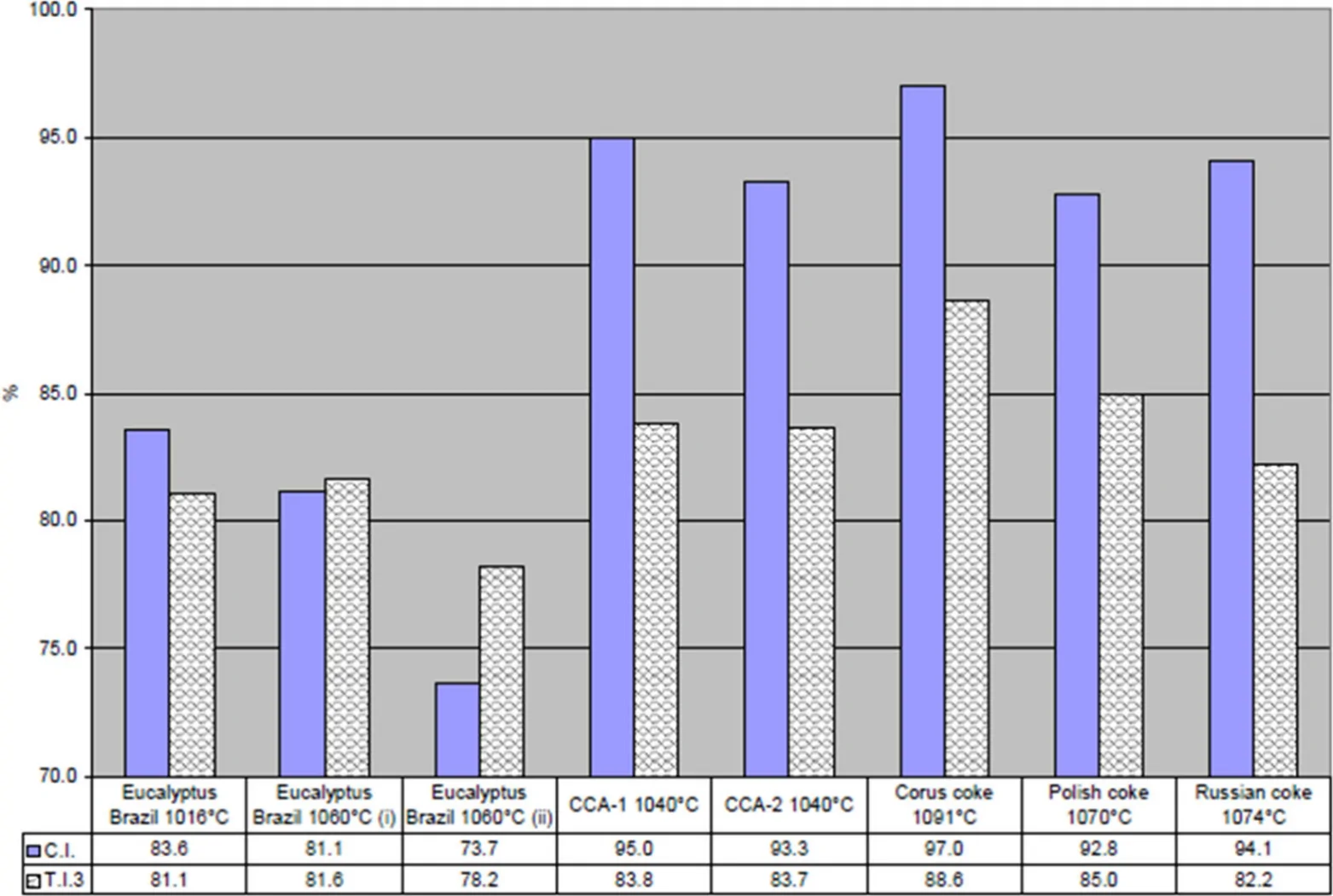
Cohesion and thermal abrasion strength of Brazilian charcoals made from Eucalyptus, preserved-wood charcoals (CCA), and metallurgical cokes (Corus, Polish, and Russian). C.I. = cohesion index and T.I.3 = thermal stability index; particles larger than 3.33 mm after tumbling. Reproduced with permission from ref 70. Copyright 2007 Mintek.

Jayakumari et al.^20^ reported that the thermal abrasion strength is slightly different between CO_2_-reacted charcoal, coal, char, and coke. However, charcoal exhibited lower cohesion strength (67%) compared to other carbon materials (87–91%). For biocokes, the cohesion strength ranged from 89 to 96%, while the thermal abrasion strength of low-density coke with 20% raw hazel shell was approximately 72%, which was significantly lower than other biocokes. Overall, most biocokes demonstrated comparable cohesion strength to coal and char, with a slight reduction in thermal abrasion strength. These results indicate that biocokes are as thermally stable as coal and char. To conclude the findings, the cohesion strength and thermal stability index of charcoal is lower than coal and cokes.

#### 2.1.6 Electrical Resistivity

During silicon and ferrosilicon production, heat is generated from the electrical energy supplied through carbon electrodes. While most of the electric current is transmitted through the electric arc, the rest flows through the reacted charge materials in the furnace.^72^ The extent of current flow through these materials is governed by their electrical resistivity. Materials with high electrical resistivity increase heat generation, leading to reduced energy consumption.^73^ The paths taken by the electric current dictate how the electrical energy (heat) is distributed throughout the furnace.^74,75^ Therefore, the electrical resistivity of carbon reductants is a crucial factor in silicon production, as it influences the energy input and the temperature profile within the furnace.^70,76–81,21,82^ The electrical resistivity of carbon materials depends on several factors such as temperature, compression pressure, particle size, and volume fraction.^78^ It is measured using a four-probe point measurement system developed at NTNU/SINTEF installed in an induction furnace (Inductotherm Europe, Droitwich Spa, UK) shown in Figure 7.^78^ The measured voltage between the wires (*V*), cross-sectional area of the sample­(*A*), the applied current (*I*), and the distance between the Mo-wires (*l*) are used to calculate the electrical resistivity, according to eq 12.

12 $$\rho =\frac{V\times A}{I\times l}$$

**Figure 7:**
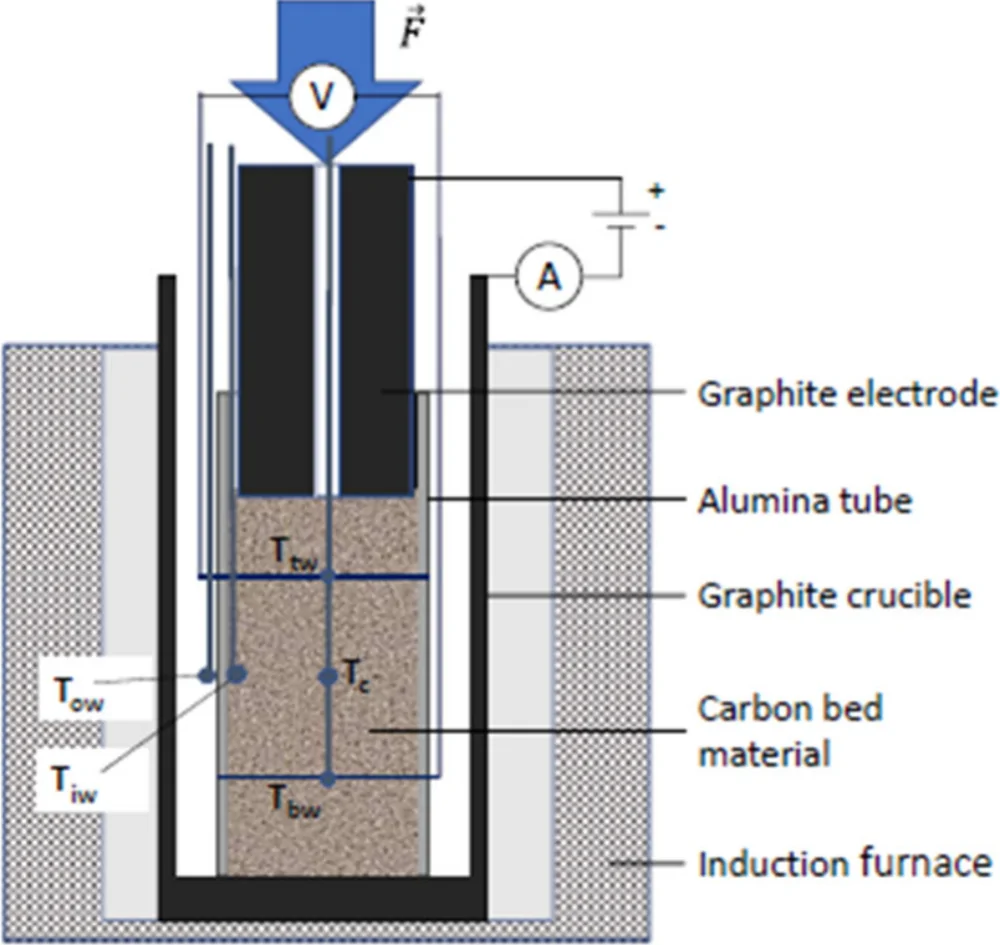
Schematic diagram of the electrical measurement test at NTNU/SINTEF. Reproduced with permission from ref 78. Copyright 2020 MDPI.

Monsen et al.^70^ reported that the electrical resistivity of various carbon materials decreased with the increase in both temperature and particle size, as shown in Figure 8. The electrical resistivity of charcoal particles (5–35 mm) at 1400–1600 °C ranged from 0.009 to 0.018 Ωm, which was approximately two or three times higher than that of coke (0.003–0.009 Ωm for 10–20 mm). Surup et al.^78^ also found that the electrical resistivity of charcoal is in a similar range of coal and metallurgical coke above 1400 °C. Overall, the electrical resistivity of carbon materials decreased with the increasing temperature, bulk density, and compaction load but the particle size has minor effects on it.

**Figure 8:**
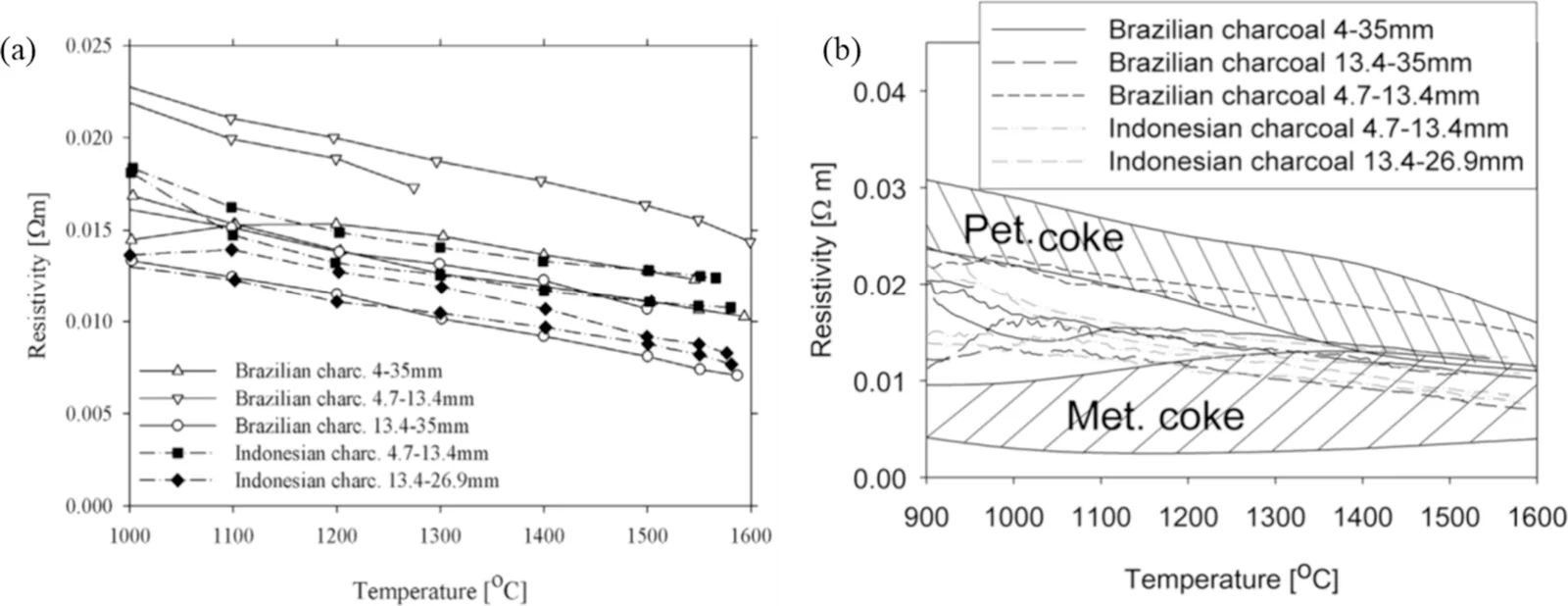
Electrical resistivity of carbon materials (a) as a function of particle size and (b) as a function of temperature. Reproduced with permission from ref 70. Copyright 2007 Mintek.

Hoover et al.^81^ compared the electrical resistivity of partially SiC-transformed carbon materials and bulk carbon materials at temperatures between 1000 and 1600 °C. In Figure 9a, the electrical resistivity of charcoal exhibited higher resistivity than char and coal at 1600 °C. In Figure 9b, the electrical resistivity of partially SiC-transformed carbon materials varied from 8 to 40 mΩm at 1600 °C. However, SiC crust samples (industrial SiC samples investigated previously by Krokstad^83^) consist primarily of α-SiC. They exhibit lower resistivity, which is more conductive than SiC-transformed carbon materials (β-SiC). The presence of Si in the SiC-converted carbon materials lowered the electrical resistivity.^80^ Overall, the SiC-transformed materials are less conductive than carbon materials and SiC crust at elevated temperatures.

**Figure 9:**
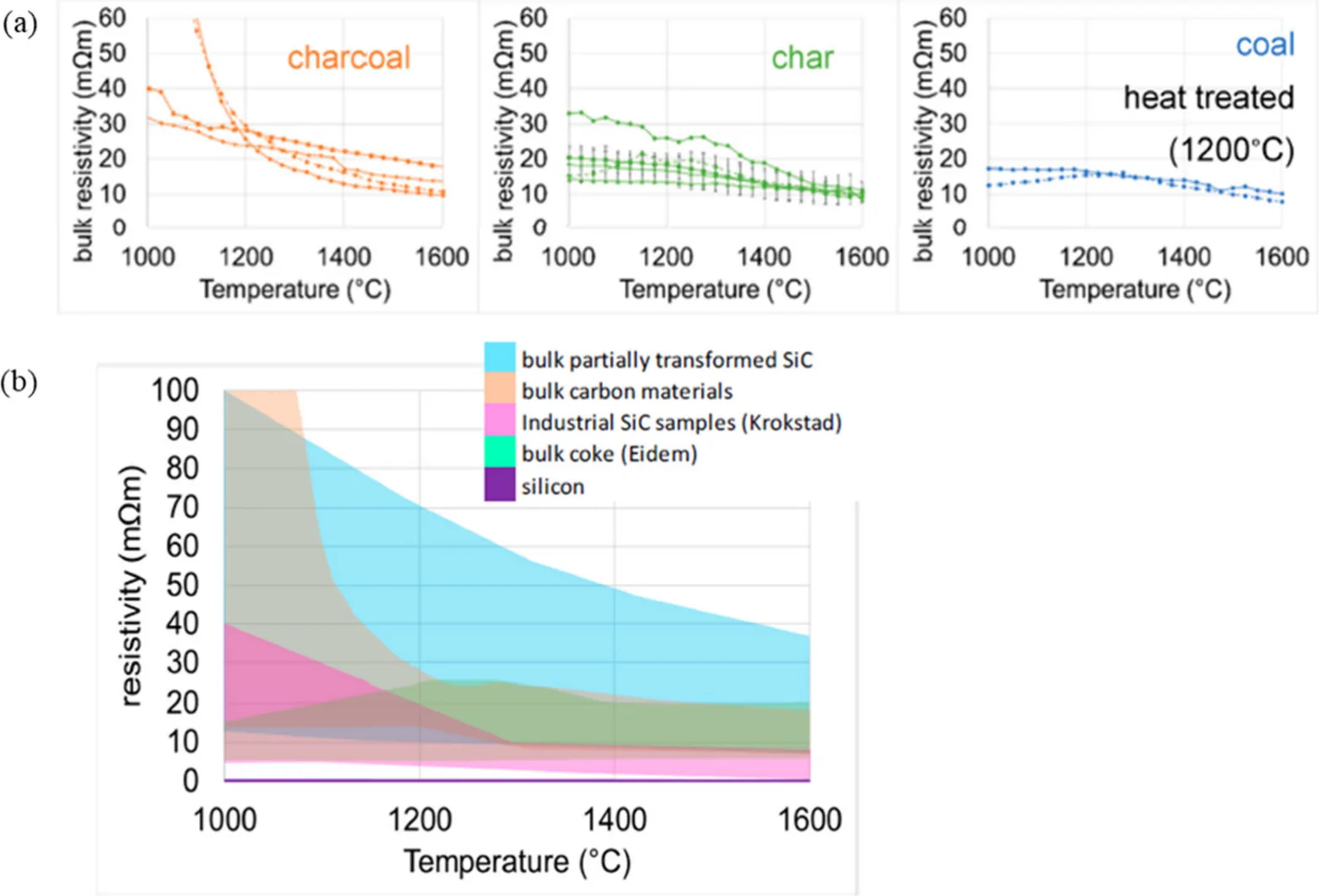
Electrical resistivity versus temperature (a) carbon materials and (b) comparison of carbon materials and SiC-converted carbon materials.^81^ (Open access).

To summarize, compared with the fossil carbon, charcoal is a renewable biocarbon and possesses superior properties such as higher porosity, greater SiO reactivity, and higher electrical resistivity. Despite these environmental and reactivity benefits, charcoal exhibits a lower bulk density as well as inferior cold and thermal strength. As a consequence, a significant amount of fines is generated during production, transportation, storage, and processing, as illustrated in Figure 10. The loss of charcoal during transportation from the pyrolysis plant to the furnace can reach up to 25%.^19^ These fines can create operational challenges related to material handling, storage, and furnace performance.^69,84^ To minimize material losses and improve resource utilization, charcoal fines can be consolidated into larger agglomerates through densification methods.^85,86^ Densification not only facilitates the recycling of charcoal fines but also improves their mechanical strength. The most common densification methods are discussed below.

**Figure 10:**
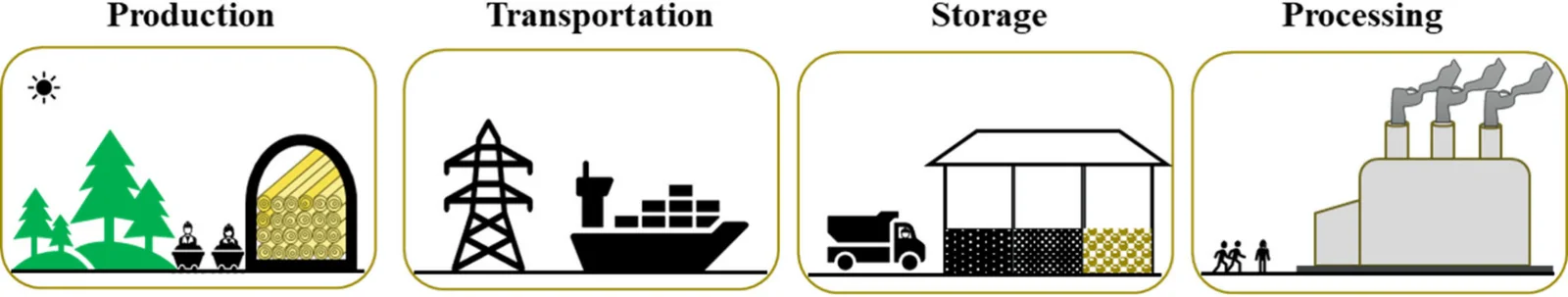
Generation of fines from charcoal from production to processing in the furnace.

### 2.2 Densification Methods

Densification is a pressure-based process that enhances cohesion between the particles by reducing void spaces, leading to an improvement of the packing density of the final product.^86,85^ The densification methods include baling, briquetting, extrusion, pelletizing, and agglomeration.^86^ Among these methods, pelletization and briquetting are the most widely used densification methods for biocarbon materials.^87,88,86,89,85^

Pelletization is a process in which the feedstock is fed into a pellet mill equipped with dies and rollers.^87,88,86,89,85^ The die and rollers forced the feedstock through the die holes, producing cylindrical agglomerates known as pellets. Pellet mills are classified into two types: ring die and flat die mills, as shown in Figure 11. In a ring-die pellet mill, the feedstock is distributed over the inner surface of a rotating, perforated die. The rollers then compressed the feedstock into the die holes. However, flat-die pellet mills are found in two types: rotating rollers where the die is stationary and a rotating die where the rollers are stationary. The feedstock descends into the chamber by gravity and is subsequently compressed between the rollers and die surface, forcing the feedstock through the die holes. The flat-die pellet mills are available in higher-capacity models, so that they experience greater wear due to variable roller travel paths. In contrast, the ring-die pellet mill is a more energy-efficient process because it generates less wear and tear due to the uniform travel paths of the rollers. Although both operate on the same fundamental principle of compressing and forcing the feedstock through die holes, pellets differ in quality and dimensions.^86,87^ The pelletization process of charcoal should not be compared to the pelletizing process of ores, which is a different process, where the pellets are produced through rolling in a ball drum or a disc.

**Figure 11:**
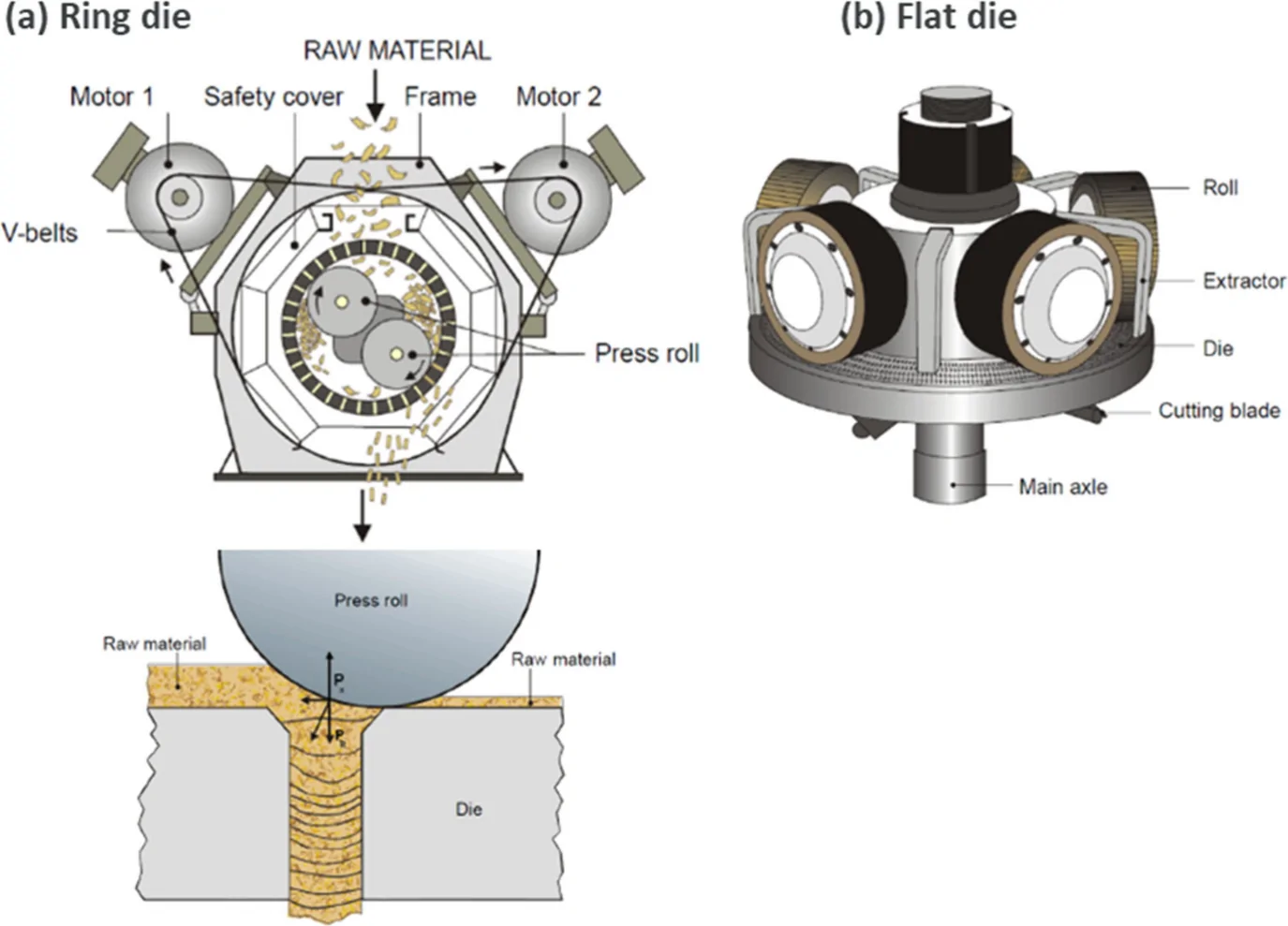
Designs of pellet mill (a) ring die and (b) flat die. Reproduced with permission from ref 87 Copyright 2002 OPET.

Briquetting is a process in which the feedstock is compacted under pressure using various types of presses, including roller press, hydraulic press, manual press, piston press, and screw press, as described in Figure 12.^90,91,85^

**Figure 12:**
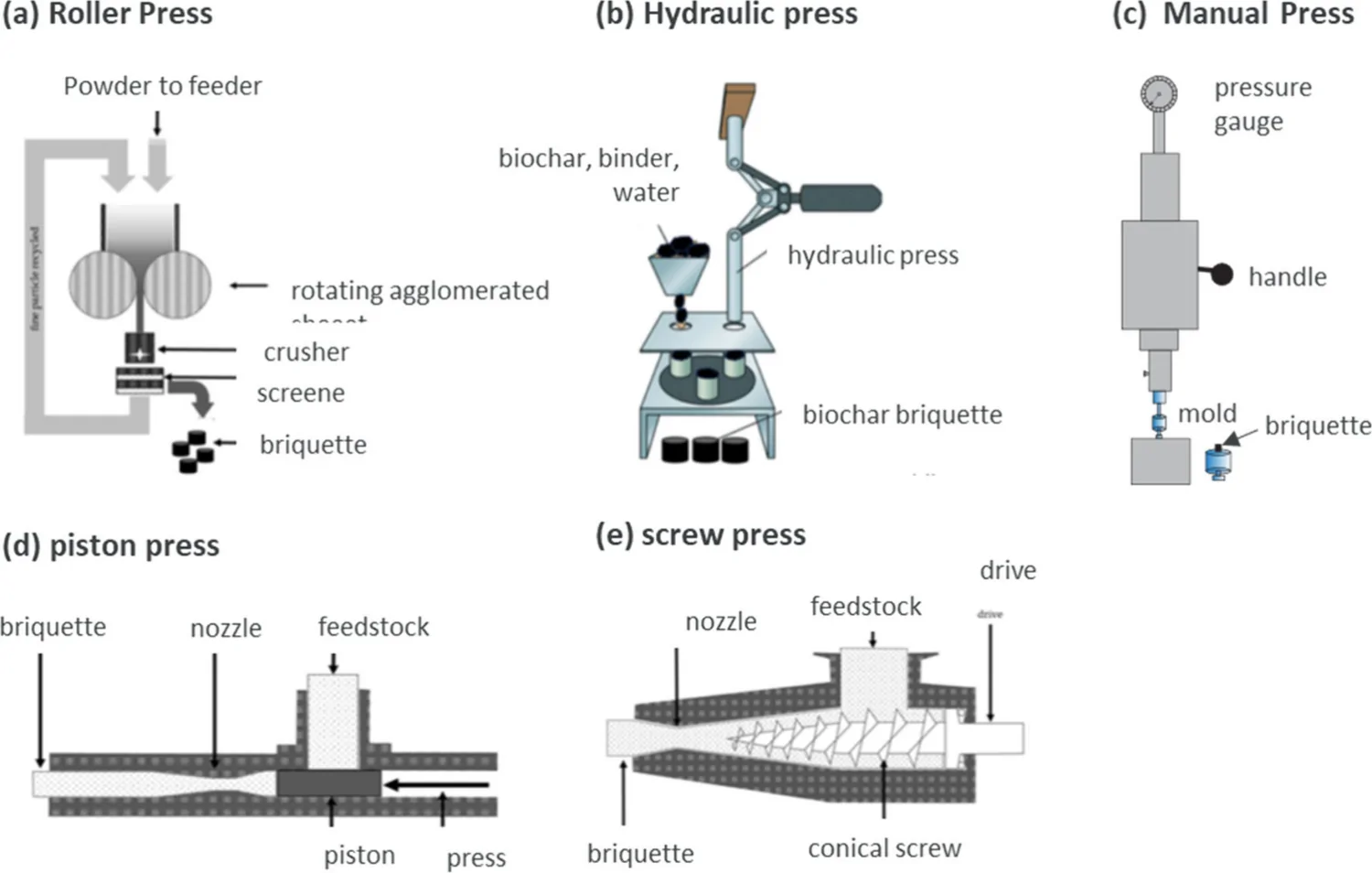
Different types of briquetting (a) roller press, (b) hydraulic press, (c) manual press, (d) piston press, and (e) screw press.^90,91,85^ (Open access).

Roller press: The feedstock is fed between two counter-rotating rollers, which compress the feedstock as it passes through.

Hydraulic press: The feedstock is compressed by a mechanical force driven by pressurized fluid from a piston.

Manual press: The feedstock was compressed using a piston operated by human effort.

Piston press: The feedstock is forced from a chamber into a die by a reciprocating ram that produces high compaction pressure.

Screw press: The feedstock is fed into a hopper and moved down through a conical compression zone where it is subjected to high pressure generated by a rotating screw.

Piston and screw presses produce briquettes with bulk densities exceeding 1000 kg/m^3^, which is higher than those produced using other types of presses.^90,91^ However, the screw press is capable of producing highly densified briquettes with bulk densities of up to 1400 kg/m^3^, leading to more energy consumption than that of the piston press.^91^ Therefore, the selection of an appropriate briquetting press depends on the desirable properties of the final briquette and energy consumption.

### 2.3 Binder

In the production of biocarbon briquettes and pellets, binders play an essential role in enhancing the cohesion between fine particles. A binder should be abundant, low-cost, pollution-free, and possess a low ash content.^92^ The binders can be divided into five groups: native or unprocessed starch, by-products from paper mills and lignocellulosic biorefineries, biosolids, inorganic materials, and fossil origin. Respective examples of each binder group are shown in Figure 13.^85^

**Figure 13:**
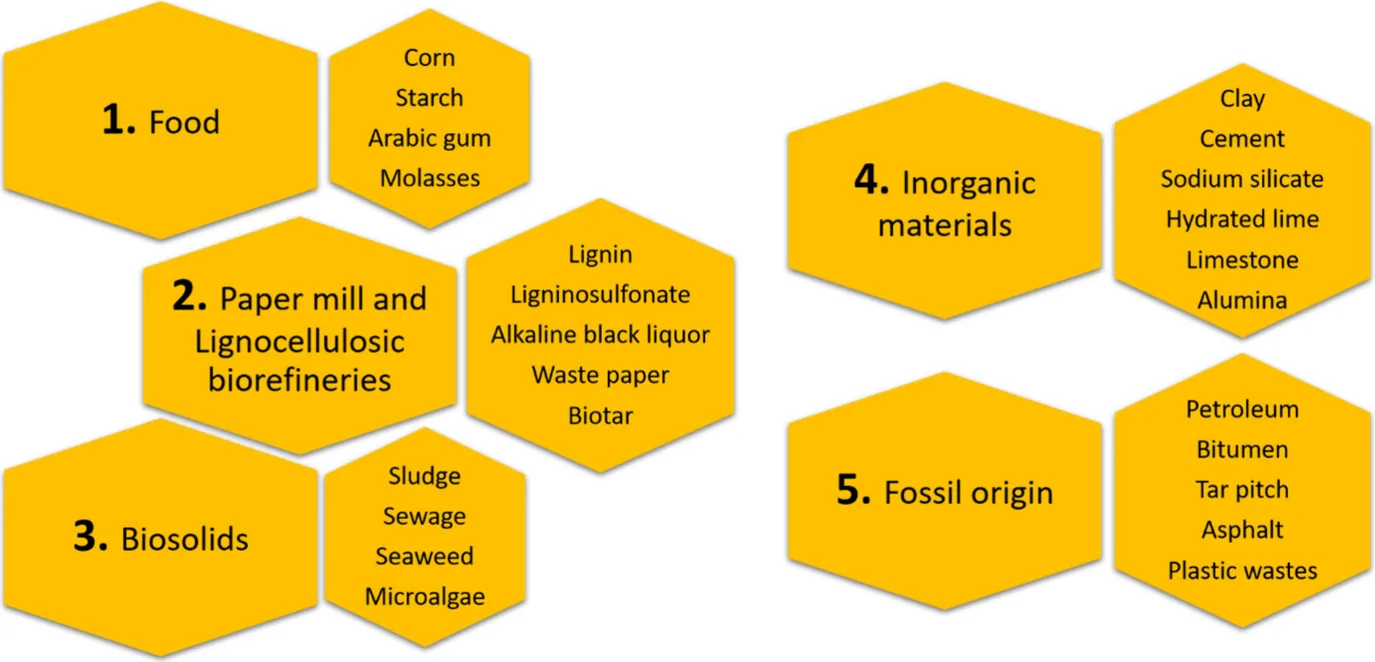
Five different groups of binders with their respective examples.

In addition, the binders are generally classified into two main types: organic and inorganic based on their nature.^85^ Briquettes prepared with organic binders exhibit high cold strength, such as tumbling strength and impact resistance index.^93^ However, organic binders readily decompose at elevated temperatures so that their mechanical strength and thermal stability are poor under high-temperature conditions. Inorganic binders possess several advantages such as high adhesion strength, nonpollution, cost-effective, and good hydrophilic properties.^93,94^ However, they do not decompose easily at high temperatures as they are noncombustible, resulting in low fixed carbon content and high ash content. A compound binder, consisting of two or more binders (organic/inorganic), offers a combination of advantages of different binders, and it has caught much attention in recent research and development.^93^

### 2.4 Charcoal Briquettes

Briquetting is used widely for the densification of charcoal fines in several studies. Table 3 summarizes binders, particle sizes, and compaction pressure from previous studies on charcoal briquettes. These parameters significantly influence the properties of briquettes. One of the common findings is that increasing the binder content and briquetting pressure enhances the adhesion between charcoal particles, thereby improving the cold strength of charcoal briquettes and reducing fines generation.^95–97^ According to the charcoal particle size, small particles (< 1 mm) increase the contact surface area between uniform particle because the binder can distribute evenly around particles, and the particles are easy to contact under briquetting pressure.^95^ The reduction in particle size also leads to lower porosity of briquettes, for instance, 1.7% porosity of 18-mesh size particles while 2.7 % porosity of 3/4 inch particles in starch briquettes.^98^

**Table 3:** Briquetting Parameters of Charcoal Briquettes Investigated in Previous Studies^a^

	briquetting parameters			
binder	binder (%)	particle size	pressure	ref
Organic				
corn starch	5–15		1 T	^96^
modified corn starch	20–40	<1–2–3 mm	15–30 MPa	^95^
starch	15	18, 5 ms, 3/4 in		^98^
industrial corn starch, pectin	5–15	2–4 mm		^97^
starch, paper, algae	10–30	2 mm		^99^
starch, paper, taro starch	10–20		1 hp	^100^
native starch. citric-acid modified starch, lignosulfonate, polyvinyl acetate, wheat flour, phenolic resin, cement, sugar syrup	5–20	0–1–3	5 MT	^101^
tapioca	2–10		40 bar	^102^
tapioca, corn starch	4–12	60 ms		^103^
tapioca starch, molasses	5–9	0.85 mm		^104^
tapioca starch, cassava peel, cassava tuber	5–20	0.3–0.7 mm	0.5 hp	^105^
cassava starch, paper, coco peat, banana peel	20–50		10 MPa	^106^
vinasse	44–60	18 ms	2.2–6.6	^107^
molasses	60–80		120 psi	^108^
polyvinyl alcohol (PVA)	1–5	<6 mm	1000 N	^109^
polyethylene terephthalate, high-density polyethylene	5–15			^110^
tannin formaldehyde resin	25–40		500 N	^111^
Inorganic				
termite mound clay	5–9	0.85		^104^
clay	15–25			^112^
clay	20–50		10 MPa	^106^
Compound				
cement, molasses, wood tar, bitumen, nanocellulose, lignin, keracoal, activator, hydrated lime, silica suspension	varies	0.5–4	160 MPa	^113^

a ms= mesh size, in = inch, T = ton

When industrial corn-starch briquettes were compared with those made with pectin, the corn-starch briquettes exhibited higher compressive strength.^97^ Modified starch briquettes demonstrated the highest compressive strength (7 MPa) and impact resistance index (>3600).^101^ However, increasing the modified starch content above 35% resulted in only marginal improvements in the compressive strength minimally, while the drop strength remained unchanged.^95^ Similarly, the addition of more than 70% molasses led to a significant reduction in the compressive strength of briquettes.^108^ These findings indicate that excessive binder addition does not necessarily provide further improvements in the cold strength of charcoal briquettes.

Charcoal briquettes produced with starch, paper and algae exhibited approximately 80% of shatter resistance. However, starch-bound briquettes showed superior water resistance (94%) compared with paper- and algae-bonded briquettes. This is due to the stability of hydrogen bonds in the starch binder, which are not readily broken by water molecules at room temperature.^99^ Narzary’s et al.^100^ similarly reported that starch- and taro-starch-bound briquettes exhibited higher shatter resistance (92%) than paper-bound briquettes. However, taro-starch-bound briquettes (68%) were more resistant to water than starch- and paper-bound briquettes.^100^ In terms of CO emissions, briquettes produced with starch generated a lower CO gas emission (223 ppm) compared with paper- and algae-bound briquettes.^99^ As the paper contains lower carbon content, paper-bound briquettes generated the lowest CO emission (2.4 g/cm^3^) compared to starch and taro-starch briquettes.^100^

Tapioca starch-bound briquettes demonstrated superior compressive strength in both vertical and horizontal directions compared with briquettes produced with cassava binders.^105^ Tapioca starch- and molasses-bound briquettes showed a very low fragility index (1.1%), whereas clay-bound briquettes showed a substantially higher fragility index of 16%.^104^ Clay-bound briquettes exhibited a high content of ash (55%) compared with briquettes produced with cassava starch, paper, coco peat, and banana peel. The ash content increased with an increasing binder content.^106,112^ In addition, due to the moisture attraction of bentonite (clay), the water resistance index of bentonite-bound briquettes decreased with increasing bentonite (clay).^114^ Clay is prone to excessive swelling upon contact with water.^115^ It does not form strong chemical bonds with charcoal surfaces, limiting its effectiveness as a binder for charcoal briquettes.

In the case of poly­(vinyl alcohol) (PVA) binder, the mechanical strength of briquettes with different proportions of PVA binder is shown in Figure 14. The compressive strength of briquettes containing 5% PVA was 115 kg/cm^2^, which was higher than lumpy charcoal (30 kg/cm^2^). However, the abrasion and shatter resistance were approximately 15-20% lower than those of the lumpy charcoal.^109^ When polyethylene terephthalate (PET) and high-density polyethylene (HDPE) were used as binder in charcoal briquettes, the compressive strength of HDPE-bound briquettes were 3–11 times higher than PET-bound briquettes.^110^

**Figure 14:**
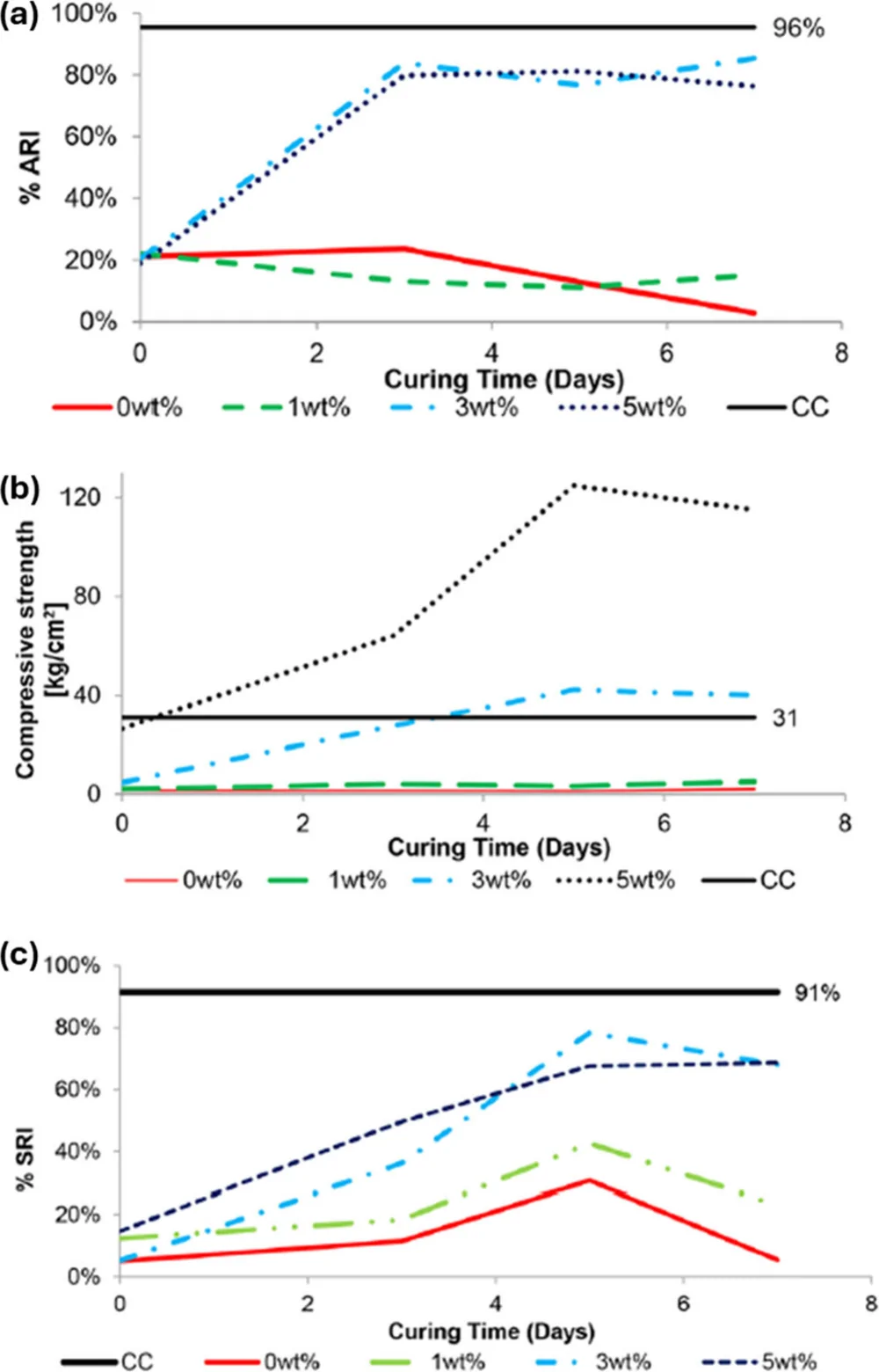
Mechanical properties of lumpy charcoal (CC) and charcoal briquettes with polyvinyl binder from 0–5 wt %; (a) abrasion resistance index, (b) compressive strength and (c) drop shatter resistance (SRI).^109^ (Open access).

The impact and shatter resistances of charcoal briquettes produced with 60% vinasse were 167 and 92%, respectively. However, they were still weaker than commercial briquettes.^107^ When tannin-formaldehyde resin (TFR) was used as a binder at concentrations ranging from 25% to 40% in charcoal briquettes, both the bulk density and impact resistance index increased up to around 74% and 1.5 g/cm^3^, respectively. The water resistance index in all briquettes remained constant at 100% because of the water-insoluble nature of the binder.^111^

Compound binders were also previously investigated in charcoal and charcoal–coal briquettes with different types of binder. In Mousa’s findings,^113^ 20% molasses-10% hydratedlime-8% cement was selected as the optimum recipe as it increased the mechanical strength of briquettes. Silica suspension addition to briquettes can reduce the surface area, resulting in a lower reactivity.

For most binders including corn starch, molasses, PET, and HDPE, increasing the binder content results in a decrease in the fixed carbon content and an increase in volatile matter in briquettes. In contrast, tapioca, taro starch, and poly­(vinyl alcohol) behave differently as the fixed carbon content tends to rise, while the ash content decreases with the binder proportion increasing.^109,100,103,104,104,102^ This contrasting trend indicates that tapioca, taro starch, and poly­(vinyl alcohol) contain less mineral content than other binders.

## 3 Conclusion

Biocarbon materials such as charcoal have the potential to replace fossil carbon reductants in silicon and ferrosilicon production because of their higher SiO reactivity. However, their mechanical properties still remain inferior to those of fossil-based carbon materials. To enhance the mechanical strength while reutilizing charcoal waste, the densification of charcoal fines with a suitable binder through pelletization or briquetting is essential. The properties of charcoal briquettes are influenced by several factors, such as binder type, compaction pressure, and particle size. With the increase in the binder content and compaction pressure, the mechanical strength of charcoal briquettes improved. However, briquettes prepared with different binders exhibit distinct behaviors because of differences in their inherent chemical composition. Despite extensive research on charcoal briquettes, their high-temperature behaviors and SiO reactivity have not yet been widely investigated. Thus, further studies on the interaction between charcoal briquettes with SiO gas at elevated temperatures are required to evaluate their suitability as biocarbon reductants for silicon and ferrosilicon production.
